# SPARC is associated with tumor nerve stroma interactions and perineural invasion in pancreatic ductal adenocarcinoma

**DOI:** 10.1038/s41598-026-51848-6

**Published:** 2026-05-11

**Authors:** Satoru Furuhashi, Ryuta Muraki, Yoshifumi Morita, Daiki Nishiwaki, Akio Matsumoto, Yuji Shimizu, Tomohiro Murakami, Makoto Takeda, Hirotoshi Kikuchi, Yoshihiro Hiramatsu, Tomoaki Kahyo, Mitsutoshi Setou, Hiroya Takeuchi

**Affiliations:** 1 Department of Surgery, Hamamatsu University School of Medicine, 1-20-1, Handayama, Chuou-ku, Hamamatsu, 431-3125 Shizuoka Japan; 2 Department of Cellular and Molecular Anatomy, Hamamatsu University School of Medicine, Hamamatsu, Japan; 3 Department of Gastroenterological Surgery, Shizuoka General Hospital, Shizuoka, Shizuoka Japan; 4 Department of Perioperative Functioning Care and Support, Hamamatsu University School of Medicine, Hamamatsu, Shizuoka Japan; 5 International Mass Imaging Center, Hamamatsu University School of Medicine, Hamamatsu, Japan; 6 Department of Systems Molecular Anatomy, Institute for Medical Photonics Research, Preeminent Medical Photonics Education & Research Center, Hamamatsu University School of Medicine, Hamamatsu, Japan

**Keywords:** Pancreatic ductal adenocarcinoma, Perineural invasion, Tumor microenvironment, Proteomics, SPARC, Biomarkers, Cancer, Oncology

## Abstract

Perineural invasion (PNI) is frequent in pancreatic ductal adenocarcinoma (PDAC) and contributes to poor prognosis, yet stromal–neural mechanisms remain unclear. We developed a vertical multi-co-culture model incorporating human PDAC cells, neonatal mouse dorsal root ganglion (DRG) neurons, and human pancreatic stellate cells (PSCs). Migration assays demonstrated markedly greater PDAC cell migration in DRG + PSC than either condition alone. Proteomic profiling of conditioned media identified Secreted Protein Acidic and Rich in Cysteine (SPARC) as a highly enriched factor in the DRG + PSC condition. Public transcriptomic datasets (TCGA, GTEx, scRNA-seq) confirmed SPARC upregulation in PDAC, with predominant expression in fibroblasts and association with poor survival. Immunohistochemistry of 81 resected PDAC specimens in Hamamatsu University School of Medicine revealed stromal-rich SPARC expression significantly correlated with PNI-positive status. Collectively, these findings indicate that stromal SPARC is associated with PNI-related aggressiveness in PDAC. SPARC may serve as a biomarker of unfavorable prognosis and as a candidate stromal factor warranting future functional validation within the tumor-nerve-stromal axis.

**Supplementary Information:**

The online version contains supplementary material available at 10.1038/s41598-026-51848-6.

## Introduction

Pancreatic ductal adenocarcinoma (PDAC) remains one of the most lethal malignancies, with a 5-year survival rate of approximately 10% globally, despite advances in diagnostic and therapeutic modalities^1^. The poor prognosis of PDAC is primarily due to the difficulty in early detection, frequent local invasion, and metastasis, making it often unresectable at the time of diagnosis. Additionally, PDAC is known for its high recurrence rate and resistance to chemotherapy^2^. Thus, improved recognition of the aggressive pathophysiology of PDAC is urgently warranted.

A distinctive feature of PDAC is its tendency for perineural invasion (PNI), with high frequency of 70.8% to 93% of surgical PDAC specimens^3,4^. The presence of PNI in PDAC is associated with local/distant recurrences and can serve as an important prognostic factor^5,6^. PNI is increasingly recognized as the fourth mode of cancer invasion, following direct invasion, lymphatic invasion, and vascular invasion^7^. During PNI progression, the nerve sheath has been proposed as the path of least resistance for tumor spreading^8^. More recently, based on neurotropic theory, the nerves and invading tumor cells can interact with each other through neurotrophins^9^. However, the detailed molecular mechanisms of PNI development in PDAC remain unclear.

The tumor microenvironment (TME), including various cellular components such as pancreatic stellate cells (PSCs, a fibroblast subset), Schwann cells, macrophages, and lymphocytes, plays a crucial role in PNI. Emerging evidence suggests that the Cancer-neuron-immune axis, involving complex cell-cell interactions mediated by growth factors and cytokines, is pivotal in the process of PNI^10^. However, the detailed mechanisms remain largely unexplored.

Thus, in this study, we sought to elucidate cell–cell interactions involved in the occurrence of PNI. Given that PDAC is characterized by an abundant desmoplastic stroma in which cancer-associated fibroblasts (CAFs) play a central role in tumor aggressiveness^11^, we hypothesized that fibroblast-lineage stromal cells may contribute to a microenvironment permissive for PNI. Based on this hypothesis, we focused on PSCs, which are well-established precursors of CAFs^12^, and established an in vitro multi-co-culture model using PDAC cells, PSCs, and nerve cells harvested from neonatal mice.

Then, the conditioned media in the multi-co-culture model were collected and protein was extracted. From proteome analysis, Secreted Protein Acidic And Rich in Cysteine (SPARC) was identified as the potential key protein to be involved in the process of PNI. SPARC protein detection and association with PNI and any clinical relevance were validated using public database and resected specimen in our institution. Our results provide new insight into the biological mechanisms underlying PNI in PDAC and identify SPARC as a candidate stromal factor associated with PNI, warranting further functional and therapeutic investigation.

## Materials and methods

### Study design and model development

**Fig. 1:**
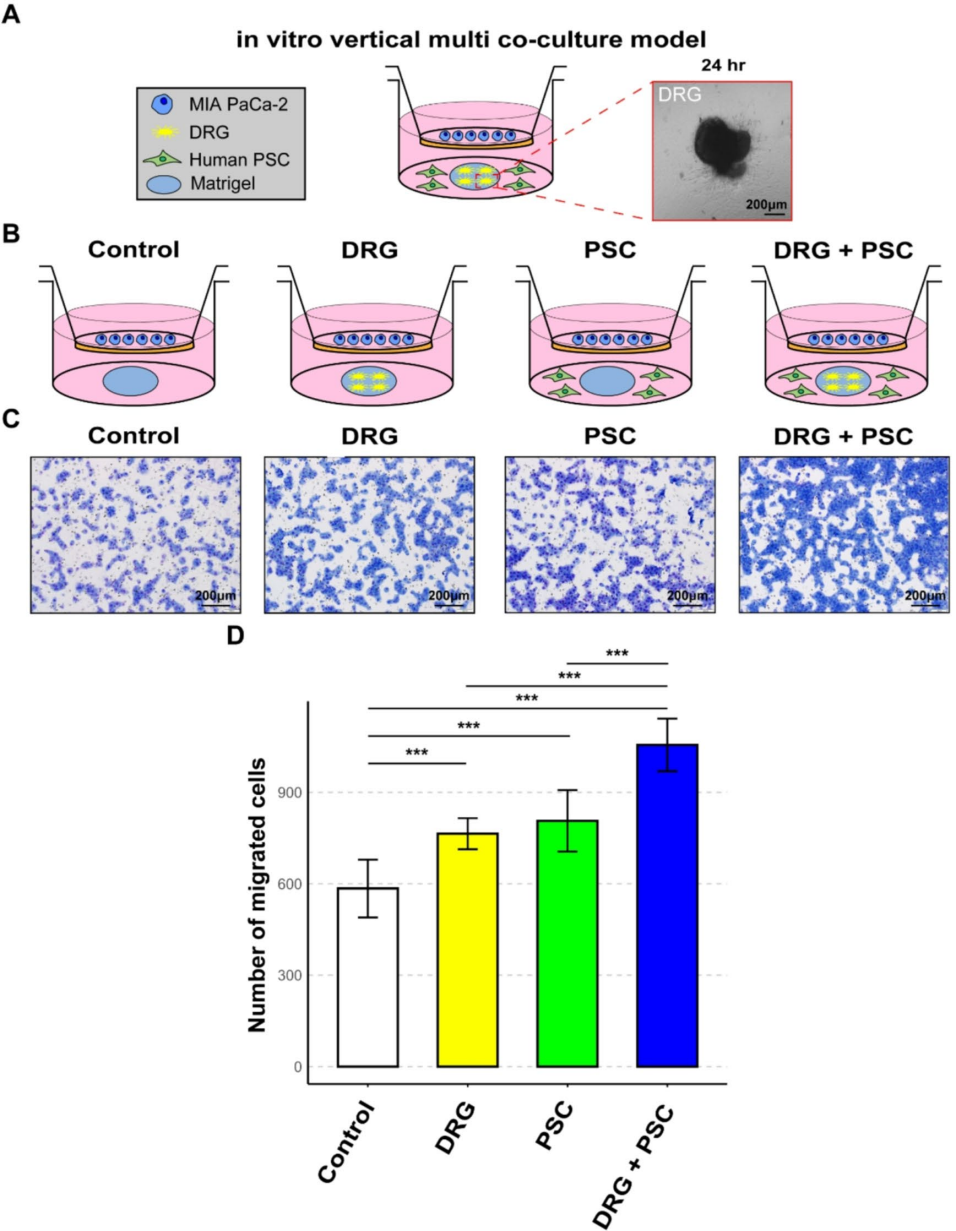
Migration of human PDAC cells in a vertical multi-co-culture model. **(A)** Schematic illustration of an in vitro vertical co-culture model in a Boyden chamber. This model consists of human pancreatic ductal adenocarcinoma (PDAC) cell line (MIA PaCa-2) seeded in the upper chamber, and dorsal root ganglia (DRGs) from neonatal mice along with human pancreatic stellate cells (PSCs), which are well-established precursors of cancer-associated fibroblasts (CAFs), placed in the lower chamber. Four DRGs were embedded in Matrigel to maintain their structural integrity. A representative image of DRG outgrowth is shown after 24 h of incubation. Scale bar = 200 μm. **(B)** Experimental setup with four conditions: Control (Matrigel only), DRG, PSC, and DRG + PSC. **(C)** Representative images of migrated PDAC cells under each condition. Cells were stained and visualized 24 h after seeding in the Boyden chamber assay. Scale bar = 200 μm. **(D)** Quantification of migrated PDAC cells under each condition. Data are presented as mean ± SD. Statistical analysis was performed using one-way ANOVA followed by Tukey’s HSD test. ***p < 0.001, PDAC, Pancreatic ductal adenocarcinoma; DRG, Dorsal root ganglion; PSC, Pancreatic stellate cell; SD, Standard deviation; HSD, honestly significant differenece.

Since 2016, our laboratory has been working to establish an in vitro co-culture model that simulates PNI process in PDAC^13,14^. This initial model co-cultured human PDAC cells with dorsal root ganglion (DRG) neurons from neonatal mice. In this study, we further developed a vertical multi-co-culture model by incorporating PSCs, thereby better recapitulating the TME and its role in facilitating PNI (Fig. 1A).

### Cell culture

A human pancreatic cancer cell line, MIA PaCa-2, was purchased from RIKEN Bioresources Cell Bank (BRC Cell Bank, Ibaraki, Japan) and provided by the Cell Resource Center for Biomedical Research, Tohoku University (Sendai, Japan). MIA PaCa-2 was routinely grown in complete Dulbecco’s modified Eagle’s medium (DMEM; Invitrogen, Carlsbad, CA, USA) supplemented with 10% Fetal Bovine Serum (FBS), at 37 °C in a humidified atmosphere saturated with 5% CO_2_. The human PSC line was purchased from ScienCell research Laboratories (No. 3830, Carlsbad, CA, USA). Human PSCs were cultured in stellate cell medium containing 2% FBS (No. 5301; ScienCell Research Laboratories).

### Isolation of DRG from neonatal mice

The following animal procedures were performed according to the guidelines of the Committee on Experimental Animals of Hamamatsu University School of Medicine (HUSM). The procedures of mouse DRG isolation were described by Ayala et al.^15^, and modified by our previous studies^11,12^. Briefly, neonatal (1-day-old) ICR mice (Japan SLC, Shizuoka, Japan) were anesthetized by isoflurane and euthanized by cervical dislocation. Each DRG was isolated by performing an anterior laminectomy and microscopic dissection from the lumbar spinal region.

### Migration assay using cancer cell-neuron vertical co-culture model

In vitro cell migration was evaluated using a cell culture insert separated by an 8-µm pore filter membrane in 24-well plates. For the cancer cell-nerve co-culture model, four DRGs collected from neonatal mice were seeded in 20 µl of Matrigel (#356231, Matrigel^®^ Growth Factor Reduced Basement Membrane Matrix, Corning, NY, USA) in the lower chamber, and incubated at 37 °C saturated with 5% CO_2_ in a humidified atmosphere for 20 min to allow Matrigel polymerization. For multi-co-culture model (cancer cell-nerve-PSC), approximately 1 × 10^4^ Human PSCs were seeded with 0.75 ml of stellate cell medium containing 2% FBS into the lower chamber. In the migration assay, 3 × 10^4^ MIA PaCa-2 cells in 0.5 ml of serum-free medium were seeded in the upper chamber. After confirmation of DRG outgrowth at 24 h, non-migrated cells remaining on the upper surface of the membrane were gently removed using cotton swabs. The migrated cells that passed through the membrane were stained with Diff Quick solution (International Reagents, Japan). Cell counting was performed in five randomly selected fields per membrane at x10 magnification under a light microscope. For each condition, four independent wells were analyzed within a single experiment and treated as technical replicates. The experiment was independently repeated three times (*n* = 3 biological replicates). The culture medium in the lower chamber was identical across all experimental conditions (Control, DRG, PSC, and DRG + PSC), consisting of stellate cell medium supplemented with 2% FBS, to eliminate potential metabolic or basal media-related confounding effects.

For Transwell cocultures with PSCs and PDAC cells, approximately 5 × 10^4^ PSCs were seeded into the lower chamber in a 6-well plate, with PDAC cells (5 × 10^4^ MIA PaCa-2 cells) growing in the top of the Transwell membrane (0.4 μm pore size; No. 3412; Corning Life Science, Tewksbury, Mass), as previously described with minor modifications^14^. PSCs cocultured with PDAC cells for 2 days were used for assays, and PDAC cells (MIA PaCa-2) monoculture and PSCs monoculture were used as controls (**Fig. S1A**).

### Western blotting

Western blotting was performed as previously described^13,14^, with slight modifications. Cells were washed with phosphate-buffered saline (PBS) and lysed in 1x Sample Buffer Solution supplemented with 2-mercaptoethanol (196-11022, FUJIFILM, Tokyo, Japan). Whole-cell lysates were denatured at 95 °C for 3 min, briefly centrifuged to remove debris, and protein concentrations were determined using a Bicinchoninic Acid (BCA) Protein Assay Kit (740967.50, TaKaRa Bio, Shiga, Japan).

Equal amounts of protein (5–10 µg per lane) were subjected to sodium dodecyl sulfate–polyacrylamide gel electrophoresis (SDS-PAGE) using 12.5% precast gels (Real Gel Plate, 13-well; SDG-538, Bio Craft, Tokyo, Japan), and subsequently transferred onto polyvinylidene difluoride (PVDF) membranes (Millipore, Burlington, MA, USA). Membranes were blocked with Blocking One solution (03953-95, Nacalai Tesque, Kyoto, Japan) for 1 h at room temperature and incubated with the appropriate primary antibodies (listed in **Table S1**) for 1 h at room temperature or overnight at 4 °C, depending on the antibody. Primary antibodies were diluted in Can Get Signal Immunoreaction Enhancer Solution (Toyobo Life Science, Osaka, Japan).

After primary antibody incubation, membranes were washed with Tris-buffered saline containing 0.1% Tween-20 (TBS-T) and incubated for 1 h at room temperature with appropriate horseradish peroxidase (HRP)-conjugated secondary antibodies (**Table S1**). Immunoreactive bands were visualized using Enhanced Chemiluminescence (ECL) Prime Western Blotting Detection Reagent (Cytiva, Marlborough, MA, USA) and visualized with a FUSION imaging system (Vilber-Lourmat, Collégien, France).

Band intensities were quantified using FUSION Edge software (Vilber-Lourmat), and the integrated density of each target protein was normalized to β-actin, which served as a loading control. Relative protein expression levels were expressed as the ratio of target protein signal intensity to β-actin signal intensity. All experiments were performed in at least four independent biological replicates. Representative cropped blot images and full-length uncropped blot images are provided in **Fig. S1B**, **Fig. S2**, and **Fig. S6**, respectively.

### Protein extraction from conditioned media in the multi-co-culture model

The collected conditioned medium from each condition (Control, DRG, PSC, and DRG + PSC; *n* = 1 per group) was processed for protein extraction and systematic identification of proteins. Initially, in the abovementioned multi-co-culture system, 20 µL of conditioned medium in the lower chamber was collected with syringe. Dithiothreitol (final concentration: 9.2 mM) was added to the sample and incubated at 40 °C for 60 min to achieve reduction. Subsequently, iodoacetamide (final concentration: 16.6 mM) was added, and the sample was incubated in the dark at room temperature for 30 min for alkylation. Trypsin (final concentration: 62.5 ng/µL; Promega, USA) was then added, and the digestion was carried out overnight at 37 °C. The reaction was terminated by the addition of 5% trifluoroacetic acid. Peptide purification was performed using a MonoSpin C18 column (GL Sciences, Inc., Tokyo, Japan). The column was preconditioned with acetonitrile, followed by equilibration with pure water. After applying the sample, the column was washed with pure water and subsequently eluted with acetonitrile. Each centrifugation step was performed at 2,500 × g for two minutes. The eluted sample was dried and reconstituted in 0.1% formic acid for downstream mass spectrometry analysis.

### Liquid chromatography–tandem mass spectrometry (LC-MS/MS) and data analysis

The sample dissolved in Solvent A (0.1% formic acid) was injected into a reversed-phase analytical column (NTCC-360/75-3-125, Nikko Technos Co., Ltd., Japan). Separation was carried out using the EASY-nLC 1000 system with a constant flow rate of 300 nL/min. Solvent B (0.1% formic acid in acetonitrile) was applied with the following gradient: 0% to 35% over 120 min, 35% to 100% over 5 min, followed by a hold at 100% for 10 min. Peptides were analyzed using a Q-Exactive™ mass spectrometer (Thermo Fisher Scientific, USA) with an electrospray ionization voltage of 1.9 kV. Full MS scans were acquired at a resolution of 70,000 over a mass-to-charge (m/z) range of 350–1800. In the data-dependent acquisition mode, the top 10 precursor ions with the highest signal intensity from each scan were subjected to higher-energy collisional dissociation with a normalized collision energy of 27%. The automatic gain control target was set at 1 × 10⁵, with a maximum injection time of 55 ms. Dynamic exclusion was applied for 20 s to prevent repeated analysis of the same ion. MS/MS data were processed using Proteome Discoverer software (version 2.2, Thermo Fisher Scientific), and peptide identification was performed by searching against the Swiss-Prot human protein database using the Mascot 2.6 search engine. A total of 85 proteins with a “high” protein false discovery rate confidence were initially identified.

After excluding proteins derived from Matrigel components and those with missing values, 51 proteins remained. Protein abundance was quantified across conditions (Control, DRG, PSC, and DRG + PSC) based on MS spectral intensity. The data were processed in R (version 4.4.1), and Z-score transformation was applied row-wise to normalize protein expression profiles. Heatmaps were generated using the pheatmap package to visualize differential protein expression among conditions. To identify proteins relevant to cellular migration, we performed a correlation analysis between migration activity across experimental conditions and protein abundance. Proteins with both (i) a Pearson correlation coefficient ≥ 0.95 and (ii) a fold change ≥ 2.0 in the DRG + PSC group compared to the Matrigel-only group were considered candidate proteins associated with enhanced migration (**Table S2**).

### Analysis of public TCGA and GTEx databases

RNA-sequencing (RNA-seq) datasets from The Cancer Genome Atlas (TCGA) and Genotype-Tissue Expression (GTEx) were downloaded from the University of California Santa Cruz (UCSC) Xena (https://xena.ucsc.edu/). The TCGA and GTEx datasets provided by UCSC Xena are uniformly processed and normalized using the Toil pipeline, allowing for direct cross-cohort comparisons^16^. For analyses within the TCGA-PDAC cohort, Illumina HiSeq RNASeqV2 data processed as RSEM-normalized counts (log2[x + 1]) were used. Although the UCSC Xena TCGA-PDAC dataset contains 183 samples, after excluding non-primary samples, 178 primary PDAC cases were included in further analyses. Using TCGA-PDAC clinical materials (*n* = 178), we manually reviewed available pathology reports and determined PNI status for 159 of the 178 cases (**Table S3**). For the institutional cohort, only patients who underwent upfront surgical resection without neoadjuvant chemotherapy were included to ensure consistent pathological evaluation. PNI was evaluated according to the Pn grading system (Pn0–Pn1c) defined in the Japanese Pancreatic Cancer Classification^17^. Assessment was performed as part of routine histopathological evaluation by experienced pathologists. To ensure consistency with the TCGA-based analysis, Pn0 and Pn1a were classified as PNI absent, whereas Pn1b and Pn1c were classified as PNI present, and PNI was analyzed as a binary variable.

### CIBERSORTx analysis

CIBERSORTx (https://cibersortx.stanford.edu/) was used to estimate cellular abundances and gene expression of each cell phenotype^18^. Single-cell RNA-sequencing (scRNA-seq) PDAC dataset (GSE111672) from the Gene Expression Omnibus (GEO) database (https://www.ncbi.nlm.nih.gov/geo/) was downloaded and applied as reference signature gene matrices following the manufacturer’s online protocol^19^. Bulk RNA-seq data from 178 PDAC patients in the TCGA cohort were analyzed using both the cell fraction mode and the high-resolution cell-type expression mode to estimate relative cellular abundances and inferred gene expression profiles within each cell type. Estimated gene expression values across all inferred cell types were log2-transformed for downstream visualization.

### Public scRNA-seq data

Publicly available single-cell RNA-seq (scRNA-seq) data were retrieved via the Tumor Immune Single-cell Hub (TISCH) database (http://tisch.comp-genomics.org/). Exploratory analyses were performed to evaluate cell-type-specific expression patterns of SPARC in human PDAC. The primary dataset analyzed was reported by Peng et al.^20^, which includes primary PDAC and non-malignant pancreatic tissues. To assess the consistency of SPARC expression patterns, two additional independent publicly available human PDAC scRNA-seq datasets (GSE111672^19^ and GSE 141017^21^ were also examined.

For each dataset, UMAP-based scatter plots of annotated cell phenotypes, uniform manifold approximation and projection (UMAP) of SPARC mRNA expression, and violin plots depicting SPARC expression across major cell populations were generated using the TISCH platform. These analyses were performed in an exploratory manner to characterize cell-type-specific expression patterns of SPARC.

### Immunohistochemistry

Immunohistochemical (IHC) staining for SPARC was performed using 4 μm-thick consecutive sections of formalin-fixed, paraffin-embedded (FFPE) tissues. The primary antibodies and dilutions employed were as follows: SPARC (ON1-1), mouse monoclonal antibody (33-5500, ThermoFisher Scientific, MA, United States) at 1:400. After deparaffinization and rehydration, samples were blocked with 3% H_2_O_2_ for 5 min at room temperature. Conditions for antigen retrieval for SPARC were performed by heating the samples at 95 °C for 20 min in citrate buffer (pH 6). Incubation duration with the primary antibody was 60 min for SPARC at room temperature. The sections were washed and then incubated with the secondary antibody (K500711, Agilent Technologies, Santa Clara, CA, United States) for one hour at room temperature. Staining signals were developed using 3,3´-diaminobenzidine (DAB, K500711, Agilent Technologies). Counterstaining was performed with hematoxylin, followed by mounting. This study was conducted in accordance with the Declaration of Helsinki and approved by the Ethics Committee of HUSM. Written informed consent was obtained from each patient. (17–323).

### Data acquisition and analysis

Whole slide image files were created by scanning the slides using NanoZoomer^®^- HT/NanoZoomer^®^2.0-HT (Hamamatsu Photonics, Hamamatsu, Japan) at 20× magnification in brightfield mode and saved as NDPi files. These files were analyzed using QuPath v0.4.3^22^. The following protocols were performed with the methodology established in our previous study^23^. Regions of interest (ROI) in the tumoral areas were established referencing the hematoxylin- and eosin-stained specimens that had been previously diagnosed by pathologists in the Department of Diagnostic Pathology. The positive cell selection feature was utilized to detect the sum of optical density (OD) for hematoxylin and DAB staining within the slides. The cell size was set within the range of 10–400 μm, with a cell expansion of 1 μm. Stromal and tumoral regions were selected as annotations, and machine learning was applied to classify objects in the ROI as tumor or stroma based on their appearance to the researcher. Thresholds for 1+, 2+, and 3 + intensities were established by selecting the mean ± standard deviation (SD) values of the compiled OD for stroma + tumor ROI. Using these thresholds (0.10, 0.25, 0.45), H-scores for stroma and tumor were calculated for the 81 slides (**Fig. S3**).

### Biostatistical analysis

All the statistical analyses were performed using the R version 4.4.1 (R Foundation for Statistical Computing, Vienna, Austria) in a two-sided way. The distribution and variation within each group of data were assessed before selecting the correct statistical analysis. Fisher’s exact test or Chi-square test was performed for comparison with nominal variables. Student’s t-test or Mann-Whitney U test was used for comparison between the two groups. Benjamini-Hochberg correction was used to decrease the False Discovery Rate (FDR). Multiple groups were compared by one- or two-way Analysis of Variance (ANOVA) followed by post-hoc tests. The correlation was determined by Pearson’s correlation test. For survival analysis using TCGA datasets, patients were divided into *SPARC*-high (SPARC-H, *n* = 139) and *SPARC*-low (SPARC-L, *n* = 39) mRNA expression groups using the minimum p-value approach^24^, with a cutoff value of 14.92. The Kaplan-Meier method and Log-rank test were used to estimate prognosis and investigate statistical significance. A logistic regression model was used for multivariate analysis. All the figures were unified using Adobe Illustrator Creative Cloud (Adobe Inc., Los Angeles, CA, United States). All data were presented as mean ± standard error of the mean (SEM) or median (range). Significant thresholds: ns (*p* ≥ 0.05), * *p* < 0.05, ** *p* < 0.01, *** *p* < 0.001, and **** *p* < 0.0001.

## Results

### Migration of cancer cells was enhanced by co-culture with DRG and PSCs in a multi-co-culture model

To evaluate the effect of the TME on PDAC cell migration, we performed an in vitro migration assay using a vertical multi-co-culture model consisting of human PDAC cells (MIA PaCa-2), DRGs (nerves), and human PSCs (stroma) (Fig. 1A). We successfully developed a vertical transwell system that permits interactions between cancer cells and DRGs or PSCs in the lower chamber (Fig. 1B). To validate the biological relevance of this model, we confirmed that PSCs exhibited increased expression of CAF-associated markers when co-cultured with PDAC cells (**Fig. S1A-C**).

Representative images of migrated cells for each condition are shown in Fig. 1C. Quantitative analysis demonstrated a significant increase in the number of migrated cells in the DRG (mean ± SD: 765.5 ± 45.3), PSC (813.2 ± 111.4), and DRG + PSC (1062.4 ± 83.9) conditions compared to the control (573.2 ± 96.8) (Fig. 1D). One-way ANOVA followed by Tukey’s HSD test confirmed significant increases in all experimental conditions compared to control (*p* < 0.001). Notably, the DRG + PSC condition induced significantly greater migration than either DRG or PSC alone, indicating a synergistic pro-migratory effect of nerves and stromal cells in this model.

### Proteomic profiling of conditioned media reveals SPARC as a candidate pro-migratory factor in the DRG + PSC co-culture microenvironment

**Fig. 2:**
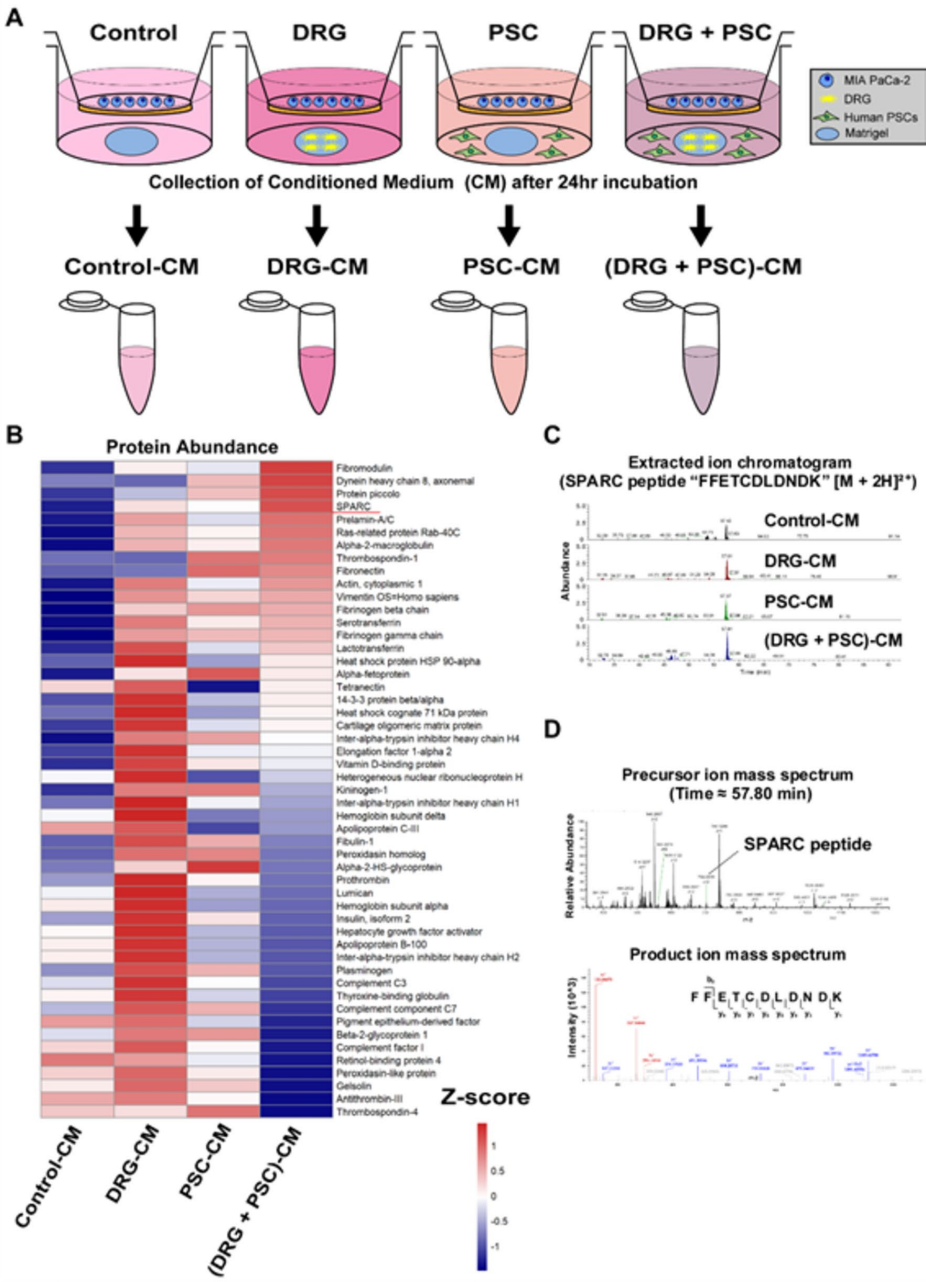
Comprehensive proteomic analysis using conditioned media from co-culture model. **(A)** Workflow of proteomic analysis. Conditioned media were collected after 24 h of co-culture under each condition (Control-CM, DRG-CM, PSC-CM, and (DRG + PSC)-CM). Proteins were extracted, digested, and analyzed by LC-MS/MS. **(B)** Heatmap of Z-score normalized protein abundance across four conditions (Control-CM, DRG-CM, PSC-CM, and (DRG + PSC)-CM). Proteins were ranked in descending order of their Z-scores in the (DRG + PSC)-CM group. Color scale represents relative protein abundance: blue indicates lower expression, white indicates intermediate expression, and red indicates higher expression. **(C)** Extracted ion chromatograms of the SPARC peptide “FFETCDLDNDK”. Signals corresponding to *m/z* 702.2928 (± 10 ppm) for the doubly charged precursor [M + 2 H]²⁺ were extracted across the four conditions. The cysteine residue was considered with carbamidomethyl modification. For comparison among conditions, the abundance scale was fixed at 5.0 × 10⁶ for all traces. **(D)** Precursor (top) and product (bottom) ion mass spectra of the SPARC peptide “FFETCDLDNDK” obtained under the (DRG + PSC)-CM condition. CM, Conditioned media; DRG, Dorsal root ganglion; PSC, Pancreatic stellate cell.

Based on the enhanced migration observed in the DRG + PSC condition, we hypothesized that soluble factors accumulating in the conditioned media may contribute to this pro-migratory microenvironment. We therefore collected conditioned media from each condition (Control, DRG, PSC, and DRG + PSC) after 24 h and performed LC-MS/MS analysis for comprehensive proteome profiling. Protein identification was performed by searching against the SwissProt database. The analysis identified 51 proteins with detectable expression in the multi-co-culture system compared with controls (**Table S2**). A heatmap based on Z-score–normalized protein abundance illustrated these differences (Fig. 2B). Based on the selection criteria (a Pearson correlation coefficient ≥ 0.95 and a fold change ≥ 2.0), SPARC (Accession No. P09486) was among the most highly enriched proteins in DRG + PSC conditioned media, prompting further evaluation in subsequent transcriptomic and histopathological analyses. Consistent with this, the extracted ion chromatogram (Fig. 2C) illustrated the relative abundance differences of the SPARC peptide “FFETCDLDNDK” across conditions, while the corresponding product ion mass spectrum, shown together with its precursor ion signal (Fig. 2D), confirmed its identification with high confidence. In parallel, PSCs exhibited increased expression of CAF-associated markers under PDAC co-culture conditions, along with a modest increase in SPARC protein levels (**Fig. S1B**,** S1C**).

### SPARC as a stromal-derived marker associated with poor prognosis in PDAC

**Fig. 3:**
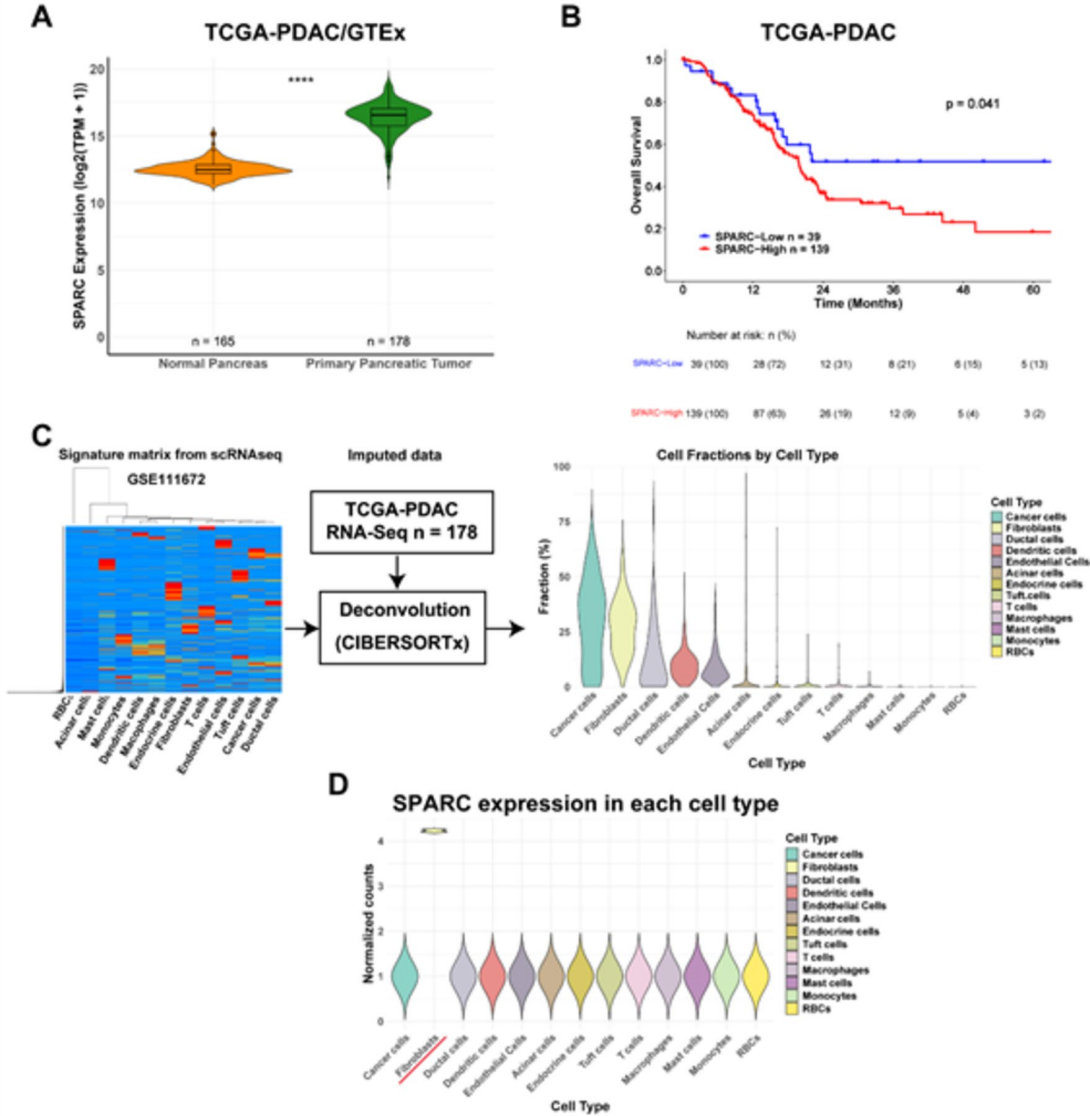
SPARC expression, prognostic relevance, and stromal localization in PDAC. **(A)** Violin plot comparing SPARC mRNA expression levels between normal pancreatic tissue (GTEx) and pancreatic tumor tissue (TCGA-PDAC). Statistical significance was determined by unpaired two-tailed t-test. ***p < 0.001. Orange = Normal Pancreas, Green = Primary Pancreatic Tumor. TPM, transcripts per million. **(B)** Kaplan–Meier overall survival curves of TCGA-PDAC patients stratified by SPARC expression (low vs. high). SPARC-high and SPARC-low groups were defined using an optimal cutpoint (minimum p-value approach; cutoff = 14.92) based on RSEM-normalized RNA-seq data (log2[x + 1]). Patients in the SPARC-high group (*n* = 139) had significantly worse overall survival compared to those in the SPARC-low group (*n* = 39). Survival differences were assessed by log-rank test (*p* = 0.041). **(C)** CIBERSORTx-based digital deconvolution of bulk RNA-seq data from 178 TCGA-PDAC patients using scRNA-seq reference profiles (GSE111672). Cell type composition and gene expression were estimated for each sample. **(D)** Inferred SPARC expression levels across major cell types based on CIBERSORTx output. Expression values represent deconvolution-based estimates rather than direct measurements, and low or undetectable expression in non-fibroblast populations may result in similar distribution patterns.

To evaluate the clinical relevance of SPARC in PDAC, we analyzed its expression using publicly available transcriptomic datasets. mRNA expression data from TCGA-PDAC and GTEx revealed that SPARC was significantly upregulated in primary pancreatic tumor tissues (*n* = 178) compared to normal pancreatic tissues (*n* = 165, Fig. 3A). Furthermore, Kaplan–Meier survival analysis based on SPARC expression levels in TCGA-PDAC demonstrated that patients with high SPARC expression (*n* = 139) had significantly worse overall survival than those with low expression (*n* = 39, log-rank *p* = 0.041, Fig. 3B). These findings suggest that elevated SPARC expression is associated with poor prognosis.

To further elucidate the cellular origin of SPARC expression in PDAC, we performed digital deconvolution analysis using CIBERSORTx. Bulk RNA-seq data from 178 TCGA-PDAC patients were analyzed using a scRNA-seq derived reference matrix generated from dataset GSE111672 (Fig. 3C). CIBERSORTx deconvolution enabled high-resolution estimation of cell composition and gene expression (Fig. 3C). The inferred expression profiles indicated that SPARC expression was enriched in fibroblast populations, including CAFs, whereas comparatively lower inferred expression was observed in cancer cells and immune cell populations **(**Fig. 3D).

Because CIBERSORTx provides model-based estimates rather than direct cell-type-specific measurements, we next examined publicly available human PDAC scRNA-data accessed through the TISCH database. Across three independent datasets, SPARC expression was consistently enriched in fibroblast populations at the single-cell resolution (**Fig. S4A-S4C**). Taken together, these findings support the interpretation that SPARC expression in PDAC is preferentially associated with fibroblast-like stromal populations.

### High stromal sparc expression positively correlates with PNI in human PDAC

**Fig. 4:**
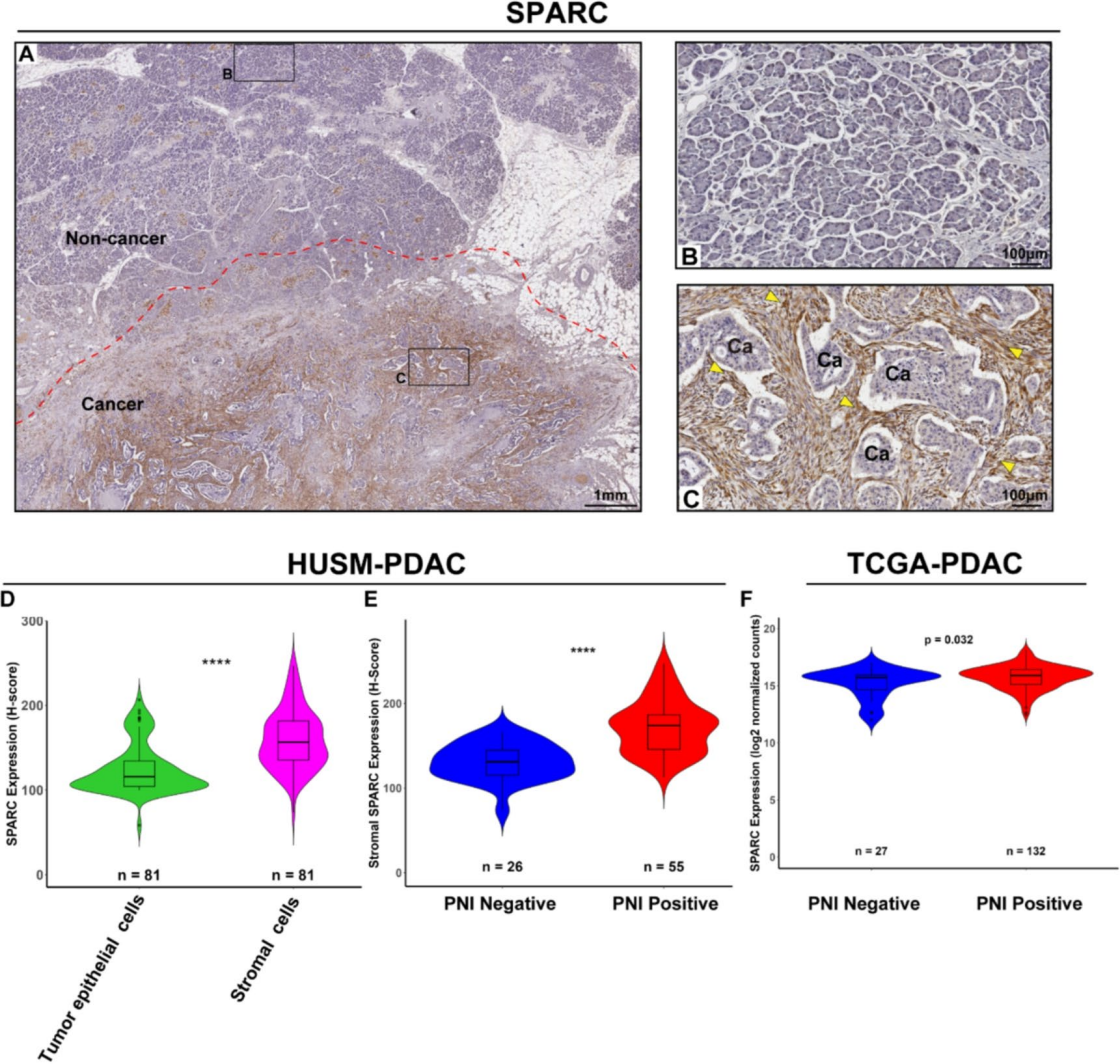
Stromal SPARC expression is associated with PNI in PDAC. **A.** Representative immunohistochemical image of SPARC protein expression in human PDAC FFPE tissue. Red dotted line indicates boundary between cancer and adjacent non-cancerous pancreatic tissues. Higher magnification views of the boxed regions in panel A are shown for adjacent non-cancerous tissue (B) and cancer tissue (C). Yellow arrowheads in (C) show SPARC-positive stromal cells surrounding cancer epithelial cells. **D.** Quantitative analysis with a semi-automated QuPath pipeline of FFPE tissues from 81 resected human PDAC cases at HUSM demonstrated that stromal compartments exhibited significantly higher SPARC protein expression compared with tumor epithelial regions. ****p < 0.0001. **E.** In the HUSM cohort (*n* = 81), stromal SPARC expression was significantly elevated in PNI-positive cases compared with PNI-negative cases. ****p < 0.0001. **F.** Validation using the TCGA-PDAC dataset showed significantly higher SPARC mRNA expression in PNI-positive tumors than in PNI-negative tumors (equal-variance t-test, *p* = 0.032).

To validate SPARC protein expression in clinical specimens, we performed IHC staining for SPARC on FFPE tissue samples from 81 PDAC patients at HUSM. SPARC was predominantly localized to the cancer stromal regions, with little to no signal in adjacent non-cancerous pancreatic tissue (Fig. 4A and C). Although focal SPARC-positive cells were occasionally observed in non-neoplastic pancreatic regions, quantitative analysis was restricted to stromal compartments within tumor areas to specifically evaluate cancer-associated stromal SPARC expression.

Quantitative assessment using a semi-automated QuPath pipeline demonstrated significantly higher SPARC levels in stromal cells than in tumor epithelial cells (*p* < 0.0001, Fig. 4D). In the HUSM cohort, stromal SPARC expression was significantly elevated in PNI Positive tumors compared with PNI Negative tumors (*p* < 0.0001, Fig. 4E). Clinicopathological characteristics in HUSM cohort according to PNI status are summarized in **Table S4**. All patients included in this analysis underwent upfront surgical resection without neoadjuvant chemotherapy. In univariate analyses, pathological T classification was significantly associated with PNI status, with more advanced tumors showing a higher frequency of PNI positivity. In addition, patients with PNI showed significantly higher stromal SPARC expression compared with those without PNI, whereas age, sex, lymphatic invasion, venous invasion, and pathological N classification were not significantly different between the groups. In logistic regression analysis, stromal SPARC expression remained significantly associated with the presence of PNI in the multivariable model, while pathological T stage also showed a trend toward association (**Table S4**). To further illustrate the spatial relationship between nerves, tumor cells, and stromal SPARC expression, representative high-magnification IHC images demonstrate that, in PNI-positive regions, SPARC expression is frequently localized to stromal cells surrounding tumor nests that are in close proximity to or encasing invaded nerves, whereas this pattern is generally not observed in PNI-negative regions (**Fig. S5**). This association was independently corroborated in TCGA-PDAC cohort, where higher SPARC expression positively correlated with PNI-positive status (*p* = 0.032, Fig. 4F), supporting the robustness of the finding. Taken together, these findings support SPARC as a stromal-derived factor strongly associated with PNI in PDAC.

## Discussion

In this study, we established an in vitro multi-co-culture system to recapitulate the cancer–neuron–stroma axis of PDAC, and identified SPARC as a stromal factor enriched under conditions associated with enhanced tumor cell migration. Transcriptomic analyses suggested that SPARC is preferentially expressed in fibroblast populations and is associated with poor patient survival. In addition, IHC analysis of resected PDAC specimens showed that stromal SPARC expression was significantly enriched in PNI-positive tumors. Collectively, these findings identify SPARC as a stromal factor associated with PNI and unfavorable prognosis in PDAC, and support its relevance within the tumor-stroma-nerve microenvironment.

Importantly, our proteomic analysis revealed that the neuron–stroma co-culture condition was enriched not only in SPARC but also in multiple ECM-associated proteins, including fibronectin and fibromodulin, which are well-established functional collaborators of SPARC^25^. These proteins are known to regulate ECM assembly, collagen organization, and cell–matrix adhesion, processes essential for cell migration and tissue remodeling^26^. Together, these findings suggest that nerve–stroma interactions induce coordinated stromal remodeling rather than isolated changes in individual proteins. In this context, SPARC is more appropriately interpreted as one component of a broader ECM remodeling program rather than a sole driver of the observed migratory phenotype. Although the present study does not directly dissect the downstream signaling pathways, the observed association supports further investigation of SPARC within the tumor-stroma-nerve microenvironment.

While SPARC has previously been implicated in ECM remodeling, EMT, and therapy resistance in various cancers, including PDAC^27–29^, its specific role in PNI remains incompletely understood. Based on prior literature, stromal SPARC may contribute to PNI through multiple mechanisms. SPARC has been reported to regulate ECM architecture and to promote matrix metalloproteinases activity, including MMP-2 and MMP-9, thereby facilitating degradation of the basement membrane and perineurium and potentially enabling perineural infiltration^30^. In addition, SPARC has been linked to integrin-mediated signaling, including focal adhesion kinase (FAK) and downstream PI3K/Akt signaling pathways, which are associated with cytoskeletal dynamics, cell motility, and neurotropic behavior of cancer cells^31^. Together, these mechanisms raise the possibility that SPARC may contribute to a microenvironment permissive for tumor cell migration and invasion, including processes relevant to perineural dissemination^32^. The interaction between CAFs and nerve fibers has been increasingly recognized as a key component of the perineural niche, and SPARC may be involved in this crosstalk as part of a broader stromal remodeling program.

Clinically, SPARC has previously been proposed as a biomarker for response to nab-paclitaxel in PDAC, although its predictive value remains controversial^28,29,33^. In this context, our findings suggest that stromal SPARC expression may serve as a biomarker of PNI-associated tumor aggressiveness. This may be particularly relevant in PDAC subtypes characterized by prominent stromal content and neural invasive features.

This study has several limitations. While our results demonstrate a strong association between stromal SPARC expression and PNI, functional inhibition experiments were not performed to establish causality. In addition, the downstream signaling pathways through which SPARC may influence the tumor microenvironment remain to be elucidated. Furthermore, spatial relationships among CAFs, nerves, and cancer cells were inferred rather than directly visualized; future studies incorporating spatial transcriptomics or multiplex immunohistochemistry will be important to clarify these interactions.

In conclusion, our findings identify stromal SPARC as a factor associated with PNI in PDAC. Rather than indicating a direct causal role, stromal SPARC may reflect a nerve-stroma-conditioned microenvironment associated with tumor aggressiveness and may serve as a biomarker for tumor-nerve-stroma interactions.

## Electronic Supplementary Material

Below is the link to the electronic supplementary material.

Supplementary Material 1

Supplementary Material 2

## Data Availability

All data supporting the findings of this study are included in the article and its Supplementary Information. De-identified raw data and analysis code are available from the corresponding author upon reasonable request.
