# Supplementary material for: SPARC is associated with tumor nerve stroma interactions and perineural invasion in pancreatic ductal adenocarcinoma
Source: Sci Rep. 2026 May 11;16:24293. doi: 10.1038/s41598-026-51848-6 (PMC13442835; doi:10.1038/s41598-026-51848-6)
Supplement: Supplementary file 1 [file 41598_2026_51848_MOESM2_ESM.docx]

**Stromal Secreted Protein Acidic and Rich in Cysteine Is Associated with Tumor–Nerve–Stroma Interactions and Perineural Invasion in Pancreatic Ductal Adenocarcinoma**

Satoru Furuhashi^1, 2*^, Ryuta Muraki^1, 2, 3*^, Yoshifumi Morita^1^, Daiki Nishiwaki^1^, Akio Matsumoto^1^, Yuji Shimizu^1^, Tomohiro Murakami^1^, Makoto Takeda^1^, Hirotoshi Kikuchi^1^, Yoshihiro Hiramatsu^1, 4^, Tomoaki Kahyo^3^, Mitsutoshi Setou^3, 5, 6^, Hiroya Takeuchi^1^

**The affiliations of the authors:**

*^1^ Department of Surgery, Hamamatsu University School of Medicine, Hamamatsu, Shizuoka, Japan*

*^2^Department of Cellular and Molecular Anatomy, Hamamatsu University School of Medicine, Hamamatsu, Japan.*

*^3^ Department of Surgery, Shizuoka General Hospital, Shizuoka, Shizuoka, Japan*

*^4^ Department of Perioperative Functioning Care and Support, Hamamatsu University School of Medicine, Hamamatsu, Shizuoka, Japan*

*^5^International Mass Imaging Center, Hamamatsu University School of Medicine, Hamamatsu, Japan.*

*^6^Department of Systems Molecular Anatomy, Institute for Medical Photonics Research, Preeminent Medical Photonics Education & Research Center, Hamamatsu University School of Medicine, Hamamatsu, Japan.*

^*^These authors contributed equally to this work.

**Address correspondence to:**

Yoshifumi Morita, MD, Department of Surgery, Hamamatsu University School of Medicine, 1-20-1, Handayama, Chuou-ku, Hamamatsu, Shizuoka, 431-3125, Japan. Phone: +81-53-435-2279, Fax: +81-53-435-2273. Email: yoshi-mo@hama-med.ac.jp

**Declaration of conflict of interest:** None.

**List of Supporting Information**

**Figure S1. Western blot analysis of CAF-associated markers and SPARC in PDAC cells and pancreatic stellate cells (PSCs).**

**A.** Schematic illustration of the experimental design, including monoculture of PDAC cells (MIA PaCa-2), monoculture of PSCs, and PSCs co-cultured with PDAC cells using a Transwell chamber system.

**B.** Representative western blot images showing protein expression levels of αSMA, FAP,

SPARC, and β-actin in PDAC cell monoculture, PSC monoculture, and PSC

co-culture with PDAC cells.

**C.** Quantification of protein expression levels (αSMA, FAP, and SPARC), expressed

as the integrated density ratio normalized to β-actin. Fold change relative to PSC

monoculture (PSC co-culture / PSC monoculture) is shown. Data represent

independent biological replicates (n = 4).

**Figure S2. Uncropped western blot images of αSMA, FAP, SPARC, and β-actin corresponding to Fig. S1.**

**Figure S3. Semi-quantitative assessment of SPARC protein expression by QuPath software**

The intensity and proportion of SPARC protein expression in tumor epithelial cells and stromal cells were semi-automatically scored. Shown are SPARC IHC images (A), tissue cell segmentation (B), and Scoring (C) performed by QuPath software.

**Figure S4**. **Single-cell analysis of SPARC expression in pancreatic ductal adenocarcinoma (PDAC) tissues using publicly available scRNA-seq datasets.**

UMAP-based scatter plots of annotated cell populations (left), SPARC mRNA expression overlaid on UMAP projections (middle), and violin plots of SPARC expression across major cell types (right) are shown for **(A)** Peng et al. (Cell Research, 2019; PMID: 31273297), **(B)** GSE111672, and **(C)** GSE141017, all derived from primary PDAC tissues and/or non-malignant pancreatic samples, with no metastatic specimens included.

The plots were generated using the publicly available TISCH platform (Tumor Immune Single-cell Hub; https://tisch.compbio.cn/) and arranged using Adobe Illustrator.

**Figure S5. Representative immunohistochemistry images demonstrating spatial localization of stromal SPRAC relative to nerves in human PDAC tissues.**

High-magnification SPARC immunohistochemistry (IHC) images are shown for

perineural invasion (PNI)-positive (A, B) and PNI-negative (C, D) PDAC cases. In

PNI-positive regions, SPARC expression is frequently observed in stromal cells

surrounding tumor nests (Ca) that are adjacent to or encasing invaded nerves (N), as

indicated by yellow arrowheads.

**Figure S6. Uncropped full-length blot images corresponding to Fig. S1.**

Uncropped full-length blots for α-SMA, FAP, SPARC, and β-actin are shown with visible membrane edges and molecular weight markers. The regions used in the **Fig. S1** are indicated by black boxes. The β-actin membrane was cut prior to antibody incubation.

**Table S1. List of antibodies (Ab) and dilutions utilized for western blotting analysis**

**Table S2. Relative abundance of secreted proteins identified in conditioned media (CM) from each condition, as measured by LC-MS/MS analysis.**

**Table S3. Distribution of perineural invasion (PNI) status across SPARC expression groups in the TCGA-PDAC cohort**

**Table S4. Clinicopathological characteristics stratified by PNI status in HUSM PDAC cohort (n = 81)**
